# Alterations of kinematics in knees after single versus multiple radius femoral prostheses total knee arthroplasty: a retrospective study

**DOI:** 10.1186/s12891-020-03425-9

**Published:** 2020-07-04

**Authors:** Mengyuan Li, Guangtao Fu, Wenhan Huang, Bofu Lin, Ruiying Zhang, Yu Zhang, Yuanchen Ma, Qiujian Zheng

**Affiliations:** 1 Division of Joint Osteopathy and Traumatology, Center of Orthopedics Surgery, Guangdong Provincial People’s Hospital, Guangdong Academy of Medical Sciences, School of Medicine, South China University of Technology, 106 Zhongshaner Road, Yuexiu District, Guangzhou, 510080 PR China; 2 Shantou University Medical College, Shantou, 515063 PR China

**Keywords:** Total knee arthroplasty, Single radius, Multiple radius, Gait kinematics

## Abstract

**Background:**

Design modifications in prostheses may cause alterations in gait kinematics, thus influencing functional restoration of knees after total knee arthroplasty (TKA). The aim of the study was to investigate the differences in gait kinematics and clinical outcomes after single radius (SR) versus multiple radius (MR) TKA.

**Method:**

The present retrospective study included 38 unilateral TKA involving 20 knees using MR design implant and 18 knees using SR design implant. Thirty-six healthy volunteers were also recruited. The mean follow-up time was 16 ± 3 months. At the end of follow-up, the 6 degrees of freedom (DOF) kinematics of knees and range of motion (ROM) were measured with a portable optical tracking system. Knee society score (KSS) and knee injury, and osteoarthritis outcome score (KOOS) were also collected.

**Results:**

Patients in the SR group had significantly higher scores in activities of daily living (84.7 ± 15.9) and sports and recreation (67.5 ± 25.2) KOOS sub-score than MR group (69.9 ± 17.6, *P* = 0.012; 50.0 ± 20.8, *P* = 0.027, respectively). Significant differences were detected between MR knees and SR knees (1.82° ± 3.11° vs 4.93° ± 3.58°, *P* = 0.009), and MR knees and healthy knees (1.82° ± 3.11° vs 3.62° ± 3.52°, *P* = 0.032) in adduction/abduction ROM. The proximal/distal translation was significantly smaller in MR knees (0.58 ± 0.54 cm) compared with SR knees (1.03 ± 0.53 cm, *P* = 0.003) or healthy knees (0.84 ± 0.45 cm, *P* = 0.039). SR knees (0.24 ± 0.40 cm) had smaller translation compared with the MR group (0.54 ± 0.33 cm, *P* = 0.017) and control group (0.67 ± 0.36 cm, *P* = 0.028). No significant difference was detected in the other DOFs during the gait cycle. Significant difference was detected in extension/flexion, internal/external rotation, adduction/abduction, proximal/distal and medial/lateral among MR, SR and healthy knees.

**Conclusion:**

After TKA, patients have altered gait kinematics compared with the control group. MR and SR design showed varied characteristics in 6 DOF gait kinematics, which could be the cause of the difference in functional outcome.

## Background

The number of total knee arthroplasty (TKA) is expected to grow exponentially in the coming years, because this procedure is effective in relieving pain and improve function in patients with end-stage osteoarthritis of the knee [1–3]. Nonetheless, approximately 20% of patients remained dissatisfied with the outcome [4]. Limited range of motion, anterior knee pain, instability and extensor insufficiency are among the leading post-operative complaints [4–6]. In vivo kinematics of the knee after TKA are alternated, and thus are probably the cause. Therefore, there has been a recent increase in recognition of the importance of the prostheses’ kinematics in accordance to the natural knee.

In the classical knee kinematics, there exist at least two transient rotating centers in the knee within the functional knee range of motion. When the femoral condyle moves downward and backward during flexion, the radius of the condylar curvature diminishes [7]. Thus, most of the femoral components of contemporary TKA are designed as multi-radius (MR). On the other hand, Eckhoff [8] reported that MR curves and changing centers of the posterior femur condyles were presented while observing from the traditional coronal, sagittal, and transverse planes. When viewing in the plane perpendicular to the transepicondylar axis, the posterior condyles of the femur were curves with single curvature radius [9]. This cylindrical axis was coincident with the natural flexion-extension axis of the knee, passing through the origins of the anterior cruciate ligament and posterior cruciate ligament, which had been confirmed by kinematics studies [7, 9]. Therefore, some femoral component designs have incorporated a single radius.

Clinical studies and meta-analysis that compared the SR and MR femoral design based on the clinical scores revealed contradictory results [4, 10–15]. The probable reason is that clinical scores may not be sensitive enough to elucidate outcome differences in implant designs. In order to highlight differences between knee prosthetic designs, demanding functional tasks are required. Our previous isokinetic and isometric data showed that SR design had advantages on higher extension and flexion strength than MR design [16]. However, knees are complex joints providing function and proprioception within 6 degrees of freedom (DOF) [17–19]. The effects of curvature modifications in design on daily activities still remain indeterminate. Walking is the most frequent activity of the daily life even for patients who had TKA. In the present study, we conducted gait analysis to assess knee kinematics during level walking. The purpose is to test the hypothesis that the kinematic behavior of the TKA knees varied with the femoral prostheses design, measuring the 6 DOF kinematics of MR or SR TKA knees.

## Methods

This retrospective, comparative study design was approved by the Institutional Review Board of our hospital. We obtained signed informed consent for participation from all study patients.

Inclusion criteria were as follows: (1) primary symptomatic osteoarthritis of the knee, patient with asymptomatic but degenerative contralateral knee was also included; (2) 55 to 85 years old; (3) body mass index (BMI) lower than 35 kg/m^2^; (4) American Society of Anesthesiologists (ASA) class 1 or 2; (5) follow-up period longer than 1 year. Exclusion criteria were as follows: (1) inflammatory arthritis, including rheumatoid arthritis, suppurative arthritis or gouty arthritis; (2) revision TKA; (3) previous tibial or femoral osteotomy; (4) flexion contracture or extension deficit more than 10°; (5) varus or valgus malalignment more than 10°; or (6) any other lower extremity disease, including tumor, infection, etc..

**Fig. 1:**
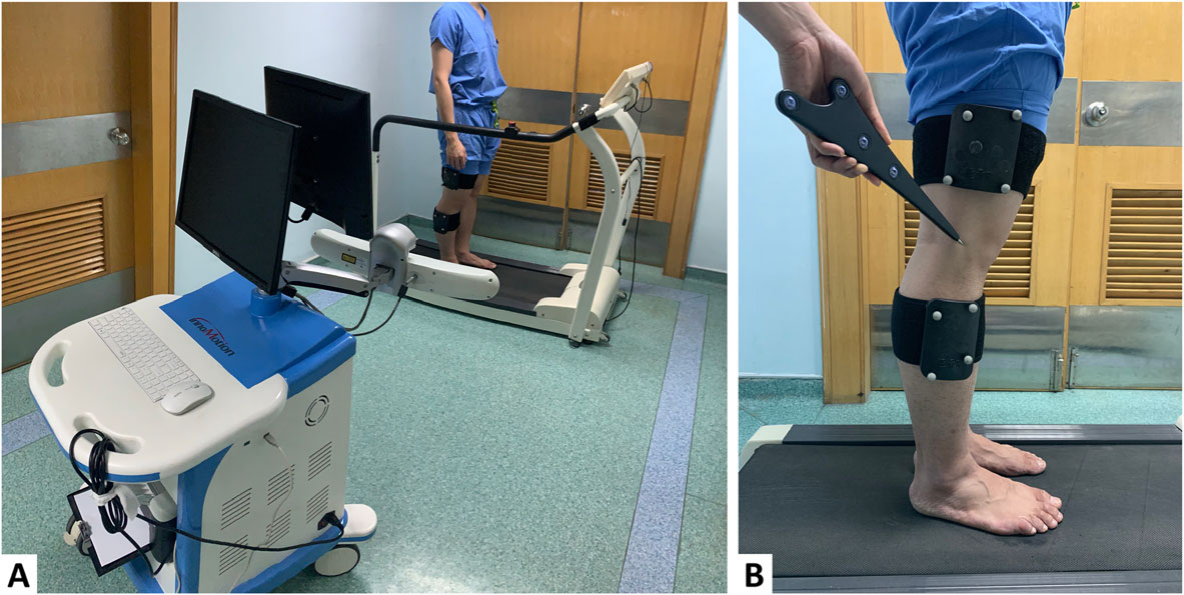
**a** The instrument for knee kinematics analysis. **b** Identification of surface markers

Knee Society Score (KSS) and Knee Injury, and Osteoarthritis Outcome Score (KOOS) were collected to assess the subjective knee function. In order to compare gait kinematics between TKA knees and normal knees, we also recruited 36 healthy people. These volunteers met the following criteria: (1) no symptomatic osteoarthritis of the knee; (2) no lower extremity deformity; (3) no other lower extremity disease, including tumor, infection, injury or history of surgery. A portable optical marker-based motion analysis system (Opti_Knee; Innomotion Inc., Shanghai, China) was utilized to measure the 6 DOF kinematics of the SR TKA knees, MR TKA knees and healthy knees during treadmill gait (Fig. 1a). The range of motion (ROM) of each DOF in the entire gait cycle was calculated. Then, comparison of 6 DOF kinematics was performed between SR and MR TKA knees. We also compared the 6 DOF kinematics between either SR or MR TKA knees and healthy knees.

The same experienced surgeon (YCM) performed the surgery. After general or spinal anesthesia and a standard pro-operative antibiotic prophylaxis, all TKAs were performed under tourniquet. The knee was exposed with a straight ventral skin incision and then a straight medial parapatellar capsular approach. The patella was not resurfaced. We applied posterior cruciate ligament (PCL)-substituting design for all cases. Measured resection technique was manually performed to achieve appropriate component alignment and patella tracking. A slight tibia posterior slope was set at 3°. For the SR group, the Stryker Triathlon TKA system (Stryker Orthopaedics, Mahwah, New Jersey) was used, and PFC sigma (DePuy Orthopaedics, Inc. Warsaw, IN, USA) was used in the MR group. On the second day, patients in both groups were encouraged to mobilize including full-weight bearing. Other post-operative exercises included continuous passive motion, active of passive knee extension.

**Fig. 2:**
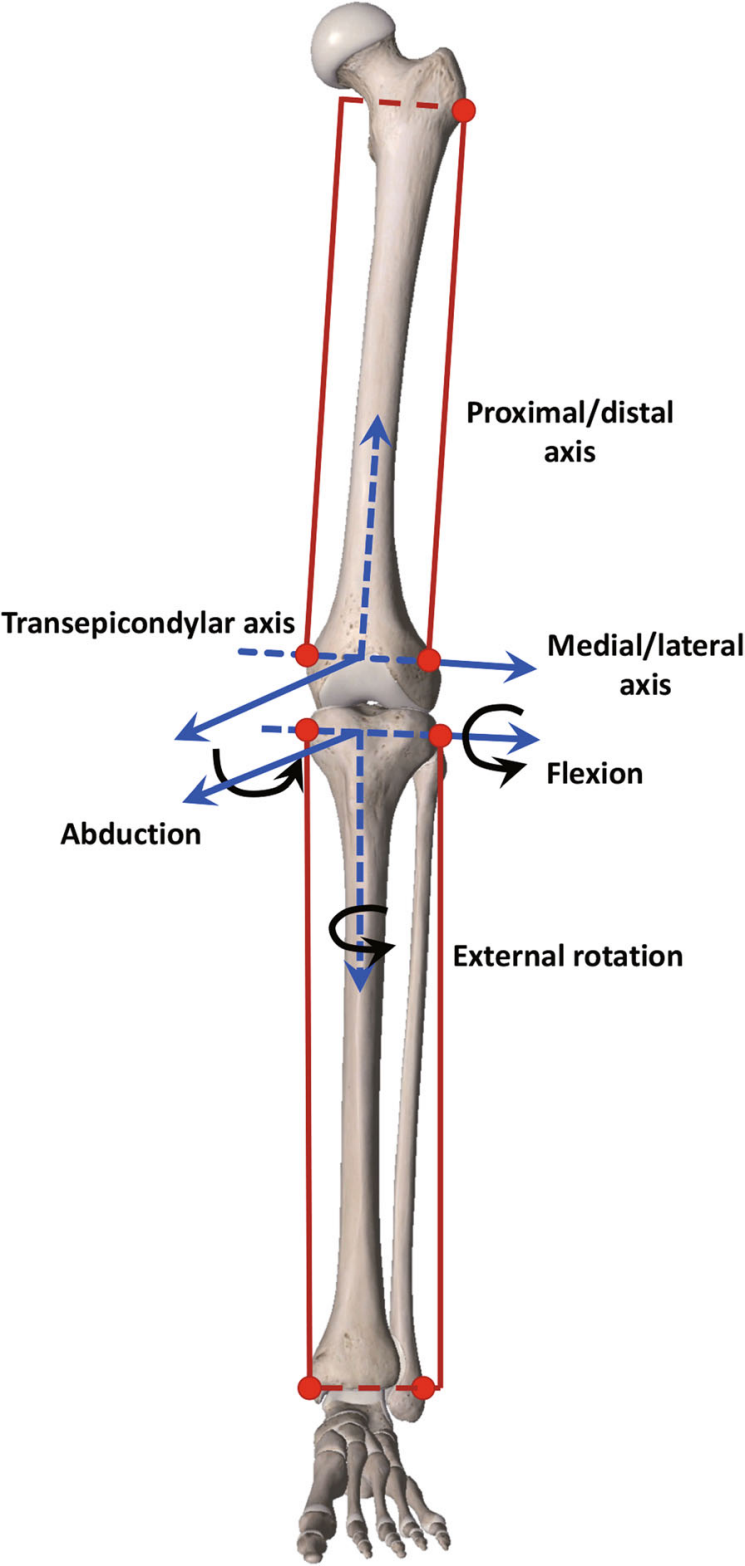
Definition of local femoral and tibial coordinate systems

Gait kinematic data was collected following a previously published protocol [17, 19]. Two rigid bodies, which comprise four infrared light-reflecting markers each (Opti_Knee; Innomotion Inc., Shanghai, China), were respectively tight onto patient’s thigh and shank with bandages. A handled digitizing probe with four infrared light-reflecting markers was used to identify greater trochanter, lateral epicondyle, medial epicondyle, lateral tibia plateau, medial tibia plateau, medial malleolus, lateral malleolus as the femoral and tibial landmarks at a static phase (Fig. 1b). Based on skeletal landmarks in the system, the femur coordinate system was built by the following steps: (1) The midpoint of the transepicondylar axis was defined as the femoral center, and a line connected the landmarks of the medial and lateral femoral epicondyles; (2) the transepicondylar line was also defined as the medial-lateral axis; (3) the anterior-posterior axis was perpendicular to the plane that was defined by the transepicondylar line and the greater trochanter; (4) the proximal-distal axis was perpendicular to the medial-lateral axis and the anterior-posterior axis. By the similar steps, we built the tibia coordinate system: (1) the center of the tibia coordinate system was located at the midpoint of the line connecting the most medial and lateral points of the tibial plateau; (2) the medial-lateral tibial plateau line was also defined as the medial-lateral axis; (3) the anterior-posterior axis was perpendicular to the plane that was defined by the medial-lateral tibial plateau line and the lateral malleolus; (4) the proximal-distal axis was perpendicular to the medial-lateral axis and the anterior-posterior axis. The rotation was defined as the orientational changes of the tibial coordinate system relative to the femur coordinate system along the anterior-posterior, medial-lateral and proximal-distal axis in the Euler angle sequence, including flexion (+)/extension, internal/external (+) rotation, adduction/abduction (+). Likewise, translation was defined as the displacement of the center of the tibial coordinate system relative to the femur coordinate system, including anterior (+)/posterior translation, proximal/distal (+) translation and medial/lateral (+) translation (Fig. 2). After a 5-min treadmill gait warm-up, the patient walked on the treadmill at a comfortable speed which resembled level-walking pattern. Subsequently, the knee was imaged for 15 s at a frame rate of 60 Hz. We applied a low-pass filter to smooth the raw kinematics data at 6 Hz, and then knee kinematics was computed and described as rotation and translation. A custom-developed MATLAB (MathWorks Inc) program was used to normalize the gait cycle from a heel strike of 0% to the next heel strike of 100% to represent a classical gait cycle. The gait cycle consists of a stance phase (0 to 62%) and a swing phase (63 to 100%). The stance phase was analyzed in 3 portions: the loading response (early stance), 0 to 12% of the gait cycle; the mid-stance, 13 to 52% of the gait cycle; and the terminal stance, 53 to 62% of the gait cycle. The swing phase was also analyzed in 3 portions: initial swing, 62 to 75% of the gait cycle; mid-swing, 76 to 85% of the gait cycle; and terminal swing, 86 to 100% of the gait cycle [17]. GraphPad Prism 8 (GraphPad Software, Inc., USA) was utilized to generate the ensemble average curve of each DOF.

### Statistical analysis

The data was described as Means and standard deviations. Kolmogorov-Smirnov test was used for testing normality. Chi-square test and Fisher’s exact test were used for categorical variables. The range of motion (ROM) in 6 DOF, KSS, KOOS were analyzed by 1-way analysis of variance (ANOVA), and the level of statistical significance was set at *P* < 0.05. The least-significant-difference (LSD) test was performed between groups when significant differences were detected. These statistical analyses were done using SPSS 26.0 (SPSS Inc., Chicago, IL, USA). A post-hoc power calculation was determined by the statistical power analyses G Power 3.1 to eliminate type II error.

## Results

### Demographics and clinical evaluation of two groups

A total of 38 patients underwent unilateral TKA were included in this study (MR group, *n* = 20; SR group, *n* = 18). The mean follow-up time was 16 ± 3 months. MR group and SR group had comparable demographic characteristics including sex, age, height, weight, body mass index, surgical side and follow-up time. The data of the two groups were considered homogeneous (Table 1). In post-hoc power calculation, the minimum α power was 0.75. Considering the small sample size of this study, we accepted α power > 0.75 for detecting a significant difference.

**Table 1:** Demographic and clinical data (mean ± SD) of included patients

	MR group (*n* = 20)	SR group (*n* = 18)	*P* value
Age, years	65.6 ± 5.8	62.7 ± 4.3	0.753
Height, m	1.58 ± 0.06	1.60 ± 0.04	0.259
Weight, kg	71.44 ± 13.13	63.64 ± 16.31	0.362
Body mass index	23.55 ± 6.93	26.31 ± 5.72	0.702
Female: male	16:4	15:3	1.000
Right: Left TKA, n	11:9	7:11	0.321
Follow-up time, months	17 ± 4	15 ± 3	0.293

*SD* Standard deviation, *SR* Single radius, *MR* Multiple radius, *TKA* Total knee arthroplasty

**Fig. 3:**
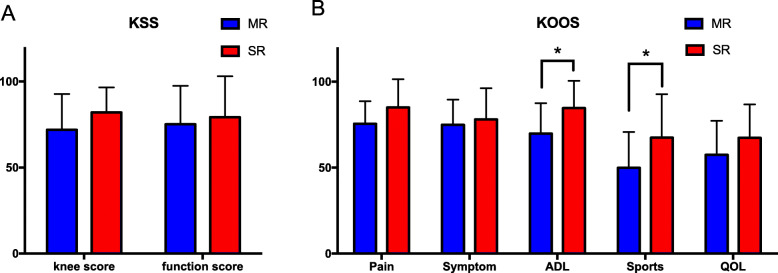
Functional outcome after TKA. Error bars denote the standard deviation of each group. Segments with significant statistical differences (*: *P* < 0.05) between the groups were marked with asterisks. (TKA: total knee arthroplasty; SR: single radius; MR: multiple radius; KSS: Knee Society Scores, KOOS: Knee Injury, and Osteoarthritis Outcome Score; ADL: activities of daily living; QOL: quality of life)

In terms of functional scores, the SR group scored significantly better in activities of daily living (84.7 ± 15.9) and sports / recreation (67.5 ± 25.2) KOOS sub-score post-operatively compared with MR group (69.9 ± 17.6, *P* = 0.012; 50.0 ± 20.8, *P* = 0.027, respectively). There were no significant differences between the two groups in other clinical outcome categories (Table 2 and Fig. 3).

**Table 2:** KSS and KOOS (mean ± SD) after TKA

	MR group	SR group	*P* value
KSS			
Knee	72.0 ± 20.7	82.1 ± 14.4	0.099
Function	75.2 ± 22.3	79.3 ± 23.8	0.595
KOOS			
1 - Pain	75.6 ± 13.1	85.1 ± 16.4	0.057
2 - Symptom	75.0 ± 14.6	78.1 ± 18.1	0.561
3 - Activities of daily living	69.9 ± 17.6	84.7 ± 15.9	0.012
4 - Sports/recreation	50.0 ± 20.8	67.5 ± 25.2	0.027
5 - Quality of life	57.5 ± 19.7	67.4 ± 19.4	0.133

*KSS* Knee Society Scores,

*KOOS* Knee Injury, and Osteoarthritis Outcome Score, *SD* Standard deviation,

*TKA* Total knee arthroplasty, *SR* Single radius, *MR* Multiple radius

### Range of motion and kinematics

**Fig. 4:**
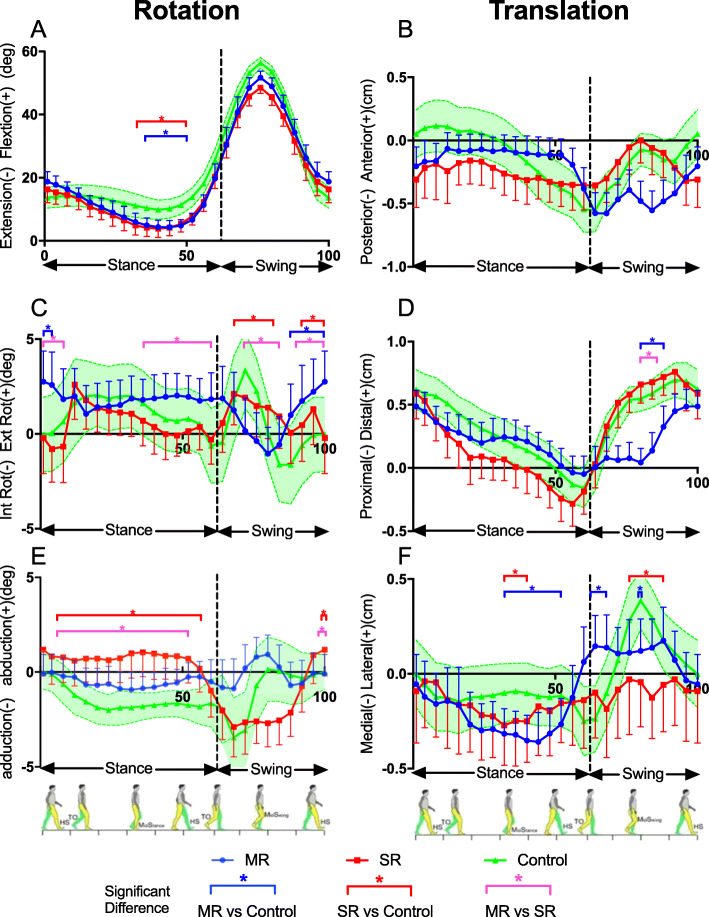
Six degree of freedom joint kinematics of control, SR, and MR knees. Average curves of knee kinematics with error bars/shadow displaying standard deviation of each group during a gait cycle. Segments with significant statistical differences (*: *P* < 0.05) between the groups were marked with asterisks. (SR: single radius; MR: multiple radius; HS: heel strike; TO: toe off)

Table 3 and Fig. 4 summarize the range of motion, translation and gait curve for MR, SR and healthy control groups.

**Table 3:** ROM (mean ± SD) among MR TKA, SR TKA and healthy knees groups

Parameters	MR	SR	Healthy	P		
				MR vs SR	MR vs healthy	SR vs healthy
Extension/Flexion ROM (°)	48.10 ± 9.03	44.23 ± 10.54	47.32 ± 7.60	0.489	0.154	0.612
Internal/External rotation ROM (°)	3.86 ± 2.33	3.41 ± 2.40	5.37 ± 4.35	0.707	0.247	0.228
Adduction/Abduction ROM (°)	1.82 ± 3.11	4.93 ± 3.58	3.62 ± 3.52	0.009	0.032	0.781
Anterior-posterior translation (cm)	0.51 ± 0.43	0.36 ± 0.54	0.54 ± 0.60	0.189	0.854	0.312
Proximal-distal translation (cm)	0.58 ± 0.54	1.03 ± 0.53	0.84 ± 0.45	0.003	0.039	0.525
Medial-lateral translation (cm)	0.54 ± 0.33	0.24 ± 0.40	0.67 ± 0.36	0.017	0.547	0.028

*SD* Standard deviation, *TKA* Total knee arthroplasty, *SR* Single radius, *MR* Multiple radius, *ROM* Range of motion

In the sagittal plane, there was no significant difference in ROM during gait cycle among MR, SR and healthy group. In regard of gait curve, significant difference was presented in MR and SR during the second half of mid-stance phase and terminal stance phase (MR: 44% ~ 58%, SR: 42 ~ 60%, respectively) compared with healthy knees (Fig. 4a).

In the axial plane, no significant difference in ROM was detected among MR, SR knees and healthy knees. Nonetheless, in the aspect of gait kinematics, MR knees showed more externally rotation during the majority of gait cycle (0 ~ 12%, 36 ~ 60%, 92 ~ 100%) than SR knees. Similarly, MR knees rotated more externally during the response phase (0 ~ 8%) and terminal swing phase (90 ~ 100%) compared with normal knees. However, during the mid-swing phase (76 ~ 84%), MR knees exhibited an internally rotating trend, while SR knees rotated in an opposite direction. SR knees showed less external rotation than healthy knees during 68 ~ 80% and less internal rotation 94 ~ 100% of gait cycle (Fig. 4c).

In the coronal plane, significant differences were detected between MR knees and SR knees (1.82° ± 3.11° vs 4.93° ± 3.58°, *P* = 0.009), and MR knees and healthy knees (1.82° ± 3.11° vs 3.62° ± 3.52°, *P* = 0.032). In the gait curve, MR knees presented more abducted mainly in stance phase than SR (8 ~ 52%) and normal (8 ~ 60%) knees (Fig. 4e).

Interestingly, although healthy knees were found to have a more obvious anterior-posterior translation (0.54 ± 0.60 cm) compared with the MR group (0.51 ± 0.43 cm, *P* = 0.003) and SR group (0.36 ± 0.54 cm, *P* = 0.039), no significant difference was detected. Moreover, no significant difference was found with regard to the gait curve (Fig. 4b).

As for proximal/distal DOF, significantly smaller translation was observed between MR knees and SR knees (0.58 ± 0.54 cm vs 1.03 ± 0.53 cm, *P* = 0.017), and MR knees and healthy knees (0.58 ± 0.54 vs 0.84 ± 0.45 cm, *P* = 0.028). In the gait curve, MR knees showed significantly less stretched than SR (78 ~ 84%) and normal knees (78 ~ 88%) in the mid-swing phase (Fig. 4d).

In medial/lateral DOF, SR knees were observed to have a smaller translation (MR: 0.54 ± 0.33 cm; SR: 0.24 ± 0.40 cm; Healthy: 0.67 ± 0.36 cm, respectively). When looking in the gait curve, the significant difference presented between TKA knees and healthy knees. For SR knees, they were significantly more medial shifted during either mid-stance phase (32 ~ 40%) and mid-swing phase (72 ~ 84%) in comparison with healthy knees. MR knees showed significantly more medial shifted during 32 ~ 48 and 80% of the gait cycle than normal knees, while during the early swing phase (62 ~ 68%) phase, MR knees were found more lateral translated than normal (Fig. 4f).

## Discussion

According to current literature, quadriceps strength and range of motion during gait decreased in patients undergoing TKAs, but other kinematic parameters such as axial rotation and anterior-posterior translation during gait were seldom analyzed because of limitations in traditional gait analysis techniques [11, 15, 20–22]. The results of our current study support the initial hypothesis that different femoral design in TKA knees can lead to varying kinematics in vivo. It is also corresponded to the clinical evaluation that patients in SR group had significantly higher scores in activities of daily living and sports / recreation KOOS sub-score than those in the MR group at the end of the follow-up.

The movements in the sagittal plane, the predominant motion during gait cycle, have been studied previously, and the flexion angles after PCL substituting TKAs during gait were decreased compared with the flexion angle of the normal [11, 15]. Nonetheless, researches compared sagittal ROM of MR and SR had contradictory results [10, 11, 15]. In this context, TKA knees, either MR or SR, had higher extension compared with control group. This could be attributed to the anterior bowing of the femur and the tibial posterior slope. As a result, the femoral and tibial components are in approximately 5° to 10° of hyperextension relative to the sagittal mechanical axis. Additionally, anterior cruciate ligament (ACL) is to limit anterior tibial translation when the knee is close to extension, so in TKA patients without ACL, they appeared to overcorrect the extended postures during the weight acceptance phase of the gait cycle in order to reduce the functional absence of ACL [23, 24].

Kinematic alterations were also identified in other DOFs in TKA knees. In the transverse plane, SR knees showed an external rotation trend within flexion. This result was in accordance with the results reported by Tamaki [15, 25]. They reported that from extension to 60° knee flexion, SR knee presented a medial pivot kinematic pattern, wherein the lateral condyle moved posteriorly significantly compared with the lesser amount of anterior-posterior translation of the medial condyle. This pivot pattern was similar to that reported in normal knee [26, 27]. Additionally, Kessler [28] discovered that the SR design showed finite helical axes concentrated on a single axis near to the medio-lateral axis of the femoral component. However, the MR design showed larger angular and spatial localization deviation, exhibiting finite helical axes varying between two axes.

With respect to the coronal plane, we found that SR knees adducted while flexion, which was close to the mode of healthy knees, but MR knees showed an opposite movement. We suspected that this phenomenon probably related to the changing radius of the femoral prostheses might change the tension on the collateral ligaments and other soft tissues at different points in the range of motion [21, 29]. So, in MR knee, the changing radius of condyle would be more likely to cause a valgus/varus rotation of the femur on the tibia, leading a perception of instability. Conversely, the SR configuration maintains the collateral ligaments in an isometric pattern during knee movement, thereby providing sustained stability.

Overall, TKA knees did not manifest a laxity more than 5 mm during active movement. We inferred that the cam-post design of tibial insert constrained the anterior translation. In the stance phase, the tibia moved posteriorly relative to femur. In the swing phase, the tibial component of SR knees tended to move posteriorly relative to femur by gravity because the tibial resection was tilted posteriorly [30]. However, in MR design the smaller radius of the femoral component at flexion angles may result in a less constrained effect in anterior-posterior direction, and furtherly lead to slight anterior translation of the femoral component [15].

As far as proximal/distal and medial/lateral translation in TKA knees, there were no such reports in the current literature. In our research, the proximal/distal translation may reflect the change of the joint gap. The compressive loading of the knee during stance phase reduced proximal/distal translation, which was consistent in two designs. In the swing phase, the joint was stretched by gravity, so the translation increased, but it was not so prominent in MR knees. This may indicate a compensation strategy that a stronger co-contraction of muscles to stabilize in the presence of mid-flexion subluxation in MR while the curvature radius changes. The range of medial/lateral translation of SR knees is less fluctuated. We considered this as another implication of the stability and isometry of collateral ligaments. It is noticed that the range of both of MR and SR groups was smaller than the control group, and it might be attributed to the lower compliance of the soft tissue around the joint after TKA.

In the current study, the difference of biomechanics parameters was in line with the difference in patient-reported outcome measures. As was discussed previously, SR knees showed better stability in the DOF of internal/external rotation, valgus/varus rotation and proximal/distal translation when comparing with MR knees. Therefore, SR knees showed higher ability in performing more exquisite activities, such as jumping, twisting and jogging. We inferred that this may be the reason for the difference in activities of daily living and sports / recreation KOOS sub-score. Similarly, Cook [12] compared 426 cases of SR and 113 cases of MR designs at 3.9-year mean follow-up and reported that the SR group had a significantly better KSSs, flexion, stability, pain, gait, and stair climbing. Sumner [31] reported that SR TKA knees had more improved kinematic during stair descent than MR TKA knees, which might reflect in the higher KSS-Function score.

This study had some limitations. First, we only included the posterior stabilized and fixed-bearing knee designs. Other designs, such as in cruciate retaining or mobilized platform ones, may mitigate the effect of radius change. Khasian [32] reported that the latest MR design, G-curve femoral prostheses, could improve stability and reduce mid-flexion paradoxical anterior sliding, but this prostheses was not included in the current study. Secondly, the small sample size of 38 and the non-consistent follow-up time point may confound the interpretation. So, a larger-sized, multi-center, long-term study is on the necessity in the future. Third, we opted for a control group of healthy subjects instead of comparing the data between the operated knee and the contralateral one, and it was difficult to match the characteristics between the healthy group and patients. Therefore, this could be a selection bias and individual walking variations could have potentially affected the outcomes. Last but not least, the retrospective design of the current study made the patient selection a confounding factor.

## Conclusion

After TKA, patients have altered gait kinematics compared with the control group. MR knees showed less adduction/abduction ROM and proximal/distal translation, while SR knees had smaller medial/lateral translation. With regard to the gait curve, both TKA knees showed more extension during stance phase. MR knees presented a varied rotating mode opposed to normal knees, and they also showed more medial/lateral displacement. SR knees partly restored the mode in internal/external rotation and proximal/distal translation when compared with normal knees, but they showed more abducted during the stance phase and more medial translated in mid-stance and mid-swing phase. These deviated characteristics in gait kinematics may be the reason for the difference in functional outcome.

## Data Availability

The data and materials used and/or analyzed during the current study are not publicly available but available from the corresponding author on reasonable request.
